# Thyrotoxic Periodic Paralysis with Hypokalemia in an Adult Male from Nepal: A Case Report

**DOI:** 10.31729/jnma.4763

**Published:** 2019-12-31

**Authors:** Sabina Khadka, Indu K.C., Rabindra Jang Rayamajhi, Pravakar Dawadi, Pravash Budhathoki

**Affiliations:** 1 Nepalese Army Institute of Health Sciences, Sanobharyang, Kathmandu, Nepal; 2 Department of Medicine, Shree Birendra Hospital, Chhauni, Kathmandu, Nepal

**Keywords:** *hyperthyroidism*, *hypokalemia*, *muscle paralysis*, *thyrotoxic periodic paralysis*

## Abstract

Thyrotoxic periodic paralysis is a rare complication of hyperthyroidism characterized by the sudden onset of hypokalemia and muscle paralysis. It is typically present in young Asian males. There are very few literatures regarding the occurrence of thyrotoxic hypokalemic periodic paralysis in Nepal. We reported a case of a 35-year-old male presented with the chief complaints of weakness of all four limbs of 1 day duration. He was diagnosed as a case of hyperthyroidism in the past, received treatment for 6 months and left medications on his own 6 months ago. Evaluation during admission revealed severe hypokalemia with serum potassium level 1.3mEq/l and high serum Triiodothyronine (>20.00μ/L) and low serum Thyroid Stimulating Hormone (<0.01μ/L). Potassium supplements resolved muscle weakness and the patient was restarted with anti-thyroid drugs. Hence, hypokalemic paralysis is a reversible cause of paralysis and high index of suspicion as well as timely interventions are required to prevent potential harm.

## INTRODUCTION

Thyrotoxic Periodic Paralysis (TPP) is potentially lethal complication of hyperthyroidism and usually presents with acute muscle weakness and hypokalemia.^1^ It is a disorder most commonly seen among Asian males.^2,3^ However, it is essential to differentiate TPP from familial hypokalemic periodic paralysis, a frequent cause of periodic paralysis in caucasians and western countries.^4^

Diagnosis is confirmed by laboratory parameters such as the presence of both hypokalemia and elevated level of thyroxine and triiodothyronine. Treatment of TPP includes correction of hypokalemia for immediate reversal of paralysis and restoration of euthyroid state for the prevention of future attacks of TPP.^5^

## CASE REPORT

A 35-year-old male presented to emergency department with a chief complaint of weakness of bilateral lower and upper limb of 1 day duration. It started at night when he tried to get up from his bed to go to the toilet. The weakness started first in the lower limbs and progressed to the upper limbs in subsequent 4 hours. The weakness was marked in the lower limbs as compared to the upper limbs. There was no history suggestive of bulbar palsy and respiratory muscle weakness. There was no history of sensory system involvement. The patient had normal bowel and bladder habit. The patient gave history of unintentional, significant weight loss of 10-15 kg over past 6 months despite normal appetite. He also had history of sweating, tremor and palpitation one year back which led to his diagnosis of hyperthyroidism 1 year back and received treatment for 6 months. He has no history of similar weakness in the past. However, he discontinued medications 6 months back on his own when his thyroid function test became normal. There was no history suggestive of similar illness in other family members.

On examination, the patient was ill looking, emaciated anxious. His vitals were:- Blood Pressure-110/40 mm of Hg, Pulse-76 beats/min, Respiratory Rate-14 breaths/ min, Temperature-afebrile. Diffuse Goiter was present. There was tremors, no exopthalmos. On Central Nervous System examination, higher mental function and the cranial nerves were found to be intact. Motor examination revealed power of 1/5 in bilateral lower limbs and power of 3/5 in bilateral upper limbs. Reflexes were absent in upper limbs and in lower limbs knee reflex was absent, however, bilateral ankle reflex was present. Sensory system was intact.

On laboratory investigation, Complete Blood Count (CBC), Renal Function Test (RFT) and Liver Function Test (LFT) reports were normal. However, the serum potassium level of the patient was 1.3mEq/l and calcium level was 7.3mg/dl. Free T3 was >20.00μg/L and TSH was <0.01μ/L. He was immediately admitted in ICU and besides cardiac monitoring, central line was placed in the patient and 100mEq potassium was given through central line over 12 hours. Injection Calcium gluconate was also given and patient was supplemented with oral calcium tablets. Within 12 hours of potassium supplementation, the potassium level corrected to 3.5mEq/l and gradually the weakness resolved. Thus, the diagnosis of thyrotoxic periodic paralysis was made. The patient was treated with Carbimazole 20mg three times a day and Propanolol 20mg four times a day and dose uptitration according to heart rate was done. Oral potassium supplementation was given.

**Figure 1:**
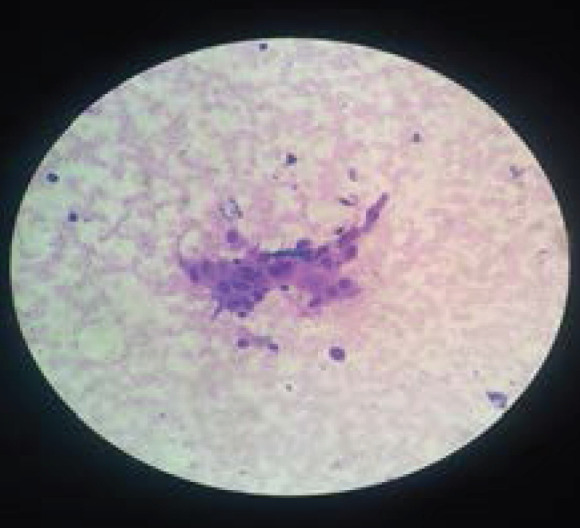
USG guided FNAC reports showing benign thyroid follicular cells in microscopic features.

The patient's USG guided FNAC reports showed benign thyroid lesion, Bethseda Category II with no atypia or evidence of malignancy and shows benign thyroid follicular cells (Figure 1). The patient's Thyroid Peroxidase Antibody (TPO Ab) was in normal range. There was improvement in the health status of the patient after the disappearance of the signs and symptoms of TPP. The patient was discharged after 2 weeks and kept on regular follow up after 1 month then after 2 months and 3 monthly thereafter.

## DISCUSSION

Hypokalaemic periodic paralysis is a rare and potentially life threatening condition which can either be due to primary (familial) or secondary cause. Secondary causes include hyperthyroidism and several other conditions such as hyperaldosteronism, diabetic ketoacidosis, nephrotic syndrome, drugs, acute tubular necrosis, laxative or diuretic abuse, diarrhoea and vomiting.^6^ The majority of Thyrotoxic periodic paralysis (TPP) is seen in hyperthyroidism due to Graves’ disease, however, toxic adenoma, thyroiditis, toxic multinodular goiter, amiodarone induced thyrotoxicosis, levothyroxine intoxication and thyrotropin (TSH) producing pituitary adenoma have all been associated with TPP.^1^ Our patient with TPP had a secondary cause i.e. he was diagnosed with hyperthyroidism around 1 year back and received treatment for 6 months with poor drug compliance.

Though hyperthyroidism is more common among females, TPP is highly prevalent among young Asian males (male: female ratio of 20:1). The prevalence of TPP is higher in Chinese, Japanese, Vietnamese, Filipino, Koreans, Malays, and Indians.^5,7^ Familial hypokalemic periodic paralysis (FPP) are frequently seen in the western countries, however some cases of TPP have also been reported from USA and several other western countries due to population mobility, admixture and increasing obesity leading to insulin resistance.^6^ FPP, an autosomal dominant disorder affecting both sexes equally, is more common in caucasians relative to TPP, and usually manifests within the first two decades of life. However, TPP usually presents in the third to fifth decade of life, is sporadic in its pattern of onset (i.e., no family history of paralysis) and is always a consequence of thyrotoxicosis.^4^ The patient in our case report is a 35-year-old male with the history of hyperthyroidism. A study by R. Sinharay reports a case which is a 30-year-old Vietnamese of Chinese parentage who presented with TPP in an Asian Emergency Department in a hospital in United Kingdom.^2^ Hence, TPP is more common in the Asian descent whereas familial periodic paralysis is seen more among the western population.

TPP is a rare complication of hyperthyroidism characterized by hypokalemia and acute muscle weakness.^1-7^ The patient in our case report also presented with weakness of bilateral lower and upper limb of 1 day. The severity of muscle weakness depends on the degree of hypokalaemia.^6^ Hypokalemia is the result of intracellular shift of potassium due to increased activity of Na^+^/K^+^-ATPase pump under the influence of increased thyroid hormones.^1-5^ The activity of Na^+^/K^+^-ATPase pump is enhanced by other factors such as insulin, adrenergic stimulation, androgens or exercise, hence, these factors may predispose to an attack in TTP patients.^3^ Patients with thyrotoxic periodic paralysis have higher Na^+^/K^+^-ATPase pump activity compared to those without paralytic attacks.^4^

Sudden onset of weakness is a typical feature of TPP that starts in the proximal muscles of the lower extremities; however, it involves all four extremities in 80% of cases. The paralytic episodes are also precipitated by heavy meals, alcohol, exercise, high salt diet, stress, infections, menstruation and glucocorticoids. The old name “nocturnal palsy” has been given to TPP because most of these attacks happen at night. More common among males aged 20-40 years, it can also be seen in adolescents and children. The patient with TPP can experience acute weakness to complete paralysis and the episode may last from few hours to 3 days.^1^ Our patient presented with the acute muscle weakness of lower limbs at night which progressed to upper limbs in about 4 hours and the weakness continued for a day.

The management of TPP involves the correction of hypokalemia with potassium chloride infusion. In, most instances, less than 50mmol of KCl is needed,^1^ however, in our case 100mEq potassium was given through central line over 12 hours. Within 1 hour of potassium supplementation, the potassium level was corrected to normal and the weakness of the patient resolved. On the other hand, overzealous replacement of potassium should be prevented in order to prevent dangerous hypokalemia. Appropriate doses of oral and intravenous propranolol can alleviate TPP where KCl is not effective. However, cautious use of propranolol is required in case of a heart block because it can lead to severe bradycardia and cardiovascular collapse.^1^

The management of TPP also includes the restoration of euthyroid state for the prevention of future attacks of TPP. Most literatures suggest radioiodine ablation or thyroidectomy as the definitive treatment of hyperthyroidism for the resolution of TPP. In our case, the patient was treated with lugol's iodine and carbimazole; in a study by Garla VV et al. and Meseeha M et al., methimazole was given.^1,4^ However, the use of antithyroid medications lead to relapse in 56% of the patient within 7 months.^1^

The diagnosis of TPP is often delayed or misdiagnosed due to the rarity of the condition and the lack of awareness of the condition.^1^ In such cases, rare complications such as life-threatening ventricular arrhythmias, acute hypercapnic respiratory failure and colonic pseudo-obstruction secondary to hypokalaemia may occur, hence it is essential to diagnose and treat TPP on time.

## Consent:

**JNMA Case Report Consent Form** was signed by the patient and the original is attached with the patient's chart.

## Conflict of Interest

**None.**
